# Acute blockage of forearm supination due to flap tear of the triangular fibrocartilage disc: A case report

**DOI:** 10.1097/MD.0000000000037915

**Published:** 2024-04-19

**Authors:** Ji Woong HO, Jee Yune Kim, Young-Keun Lee

**Affiliations:** a Department of Orthopedic Surgery, Research Institute of Clinical Medicine of Jeonbuk National University – Biomedical Research Institute of Jeonbuk National University Hospital, Jeonju, Jeonbuk, Republic of Korea.

**Keywords:** arthroscopic, supination, surgeries, triangular fibrocartilage, wrist

## Abstract

**Rationale:**

Acute blockage of forearm supination has been reported in several studies. It is caused by loose bodies in the wrist joint, extensor carpi ulnaris tendon interposition, and distal radioulnar joint (DRUJ) injuries, including forearm bone fractures. Some studies have reported cases of DRUJ injuries caused by triangular fibrocartilage complex (TFCC) tears.

We report a case of acute blockage of forearm supination after minor trauma and suggest a possible TFCC tear when a patient complains of forearm supination blocking. In addition, we present a comparison between our case and other reports on etiology, magnetic resonance imaging (MRI) findings, and arthroscopic findings, and show the specific characteristics of our case.

**Patients concerns:**

A 22-year-old male presented with left wrist pain as the chief complaint. He was injured 2 months prior to pushing his left hand on the floor during exercise. Physical examination showed a relative limitation of range of motion (ROM) in the left wrist of about 10° in flexion and about 15° in extension compared with the right side. The patient also complained of supination limitation and volar side wrist pain during supination. The patient showed tenderness in the axial compression test.

**Diagnoses:**

Plain radiographs showing no abnormalities. MRI showed a TFCC tear in the central portion. A torn flap of the TFCC was interposed on the volar side of the DRUJ.

**Interventions:**

Arthroscopic surgery of the left wrist joint was performed. Arthroscopic examination revealed a tear in the TFCC on the radial side. A torn flap was interposed on the volar side of the DRUJ. We removed the flap from the DRUJ using an arthroscopic grasper and partially resected it.

**Outcomes:**

Intraoperative tests showed no locking and the forearm was well supinated. Two months after the surgery, the patient had no pain and showed full forearm supination.

**Lessons:**

DRUJ blocking due to a TFCC tear should be suspected when acute blockage of forearm supination occurs after minor trauma. MRI is helpful for diagnosis; however, we suggest that the diagnosis should be confirmed through arthroscopy. Symptoms can be resolved by surgical treatment using arthroscopy.

## 1. Introduction

Acute blockage of the forearm supination is relatively rare. When this occurs, it is important to accurately diagnose the causes and resolve them to provide patients with a full range of motion (ROM) of the forearm. A few causes have been reported, including loose bodies, extensor carpi ulnaris tendon interposition, and distal radioulnar joint (DRUJ) injuries including forearm bone fractures.^[1–8]^ Especially for DRUJ injuries, some studies have reported that after minor trauma, patients showed forearm supination blocking. This blocking occurs mainly in young patients, and although supination is limited, pronation is possible in these patients.^[1,2]^ Studies have suggested that blockage due to the triangular fibrocartilage complex (TFCC) flap during ulnar head translation is a possible mechanism.^[1–6]^ Therefore, patients with blockage of forearm supination caused by TFCC rupture must be checked for abnormalities in the bony structure, including the ulnar head, through radiological examinations such as plain radiography and computed tomography. The presence of TFCC injury can be confirmed through magnetic resonance imaging (MRI) and arthrogram.^[1]^

In addition, the diagnosis can be confirmed through arthroscopy by checking whether a ruptured flap of the TFCC causes DRUJ blockage. The flap could be removed using an arthroscopic technique. We report our experience in diagnosing DRUJ blocking due to TFCC flap tear through MRI and arthroscopic examination, and resolving symptoms through arthroscopic treatment in a patient who complained of acute forearm supination blocking after minor trauma.

Additionally, we suggest some specific characteristics of our patient etiology, imaging findings, and arthroscopic findings compared with other reports.

### 1.1. Consent

The patient signed an informed consent form for the publication of this case report and any accompanying images. Ethical approval for this study was waived by the ethics committee of Jeonbuk National University Hospital because it was a case report and there were fewer than 3 patients (2023-11-028).

## 2. Case presentation

A 22-year-old male presented with a chief complaint of pain in the left wrist. The patient complained of pain, mainly in the supination position. He was injured 2 months ago to pushing the left hand on the floor during exercise.

**Figure 1.**
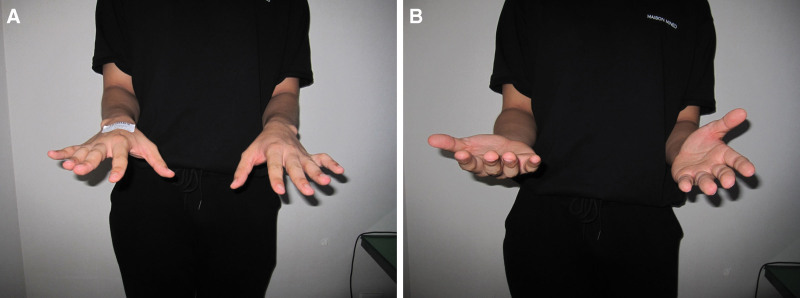
(A, B). Preoperative gross photo for range of motion (ROM) of pronation (A) and supination (B) in both forearms. It shows relatively limited supination in the left forearm compared to the right side.

Physical examination showed limitation of ROM in the left wrist by 10° in flexion and about 15° in extension compared with the right side. He also complained of supination limitation. He was able to supinate the wrist to only approximately 40° (Fig. 1A and B). During further forearm supination, the patient experienced volar side wrist pain. The grip power was diminished compared to that of the contralateral hand: 22 kg strength in the left hand and 28 kg in the contralateral hand. There were no signs of foveal tenderness or DRUJ instability; however, he showed tenderness in the axial compression test. We checked some of the scoring surveys. The initial visual analog scale score was 2 points. The Quick Disabilities of Arm, Shoulder, and Hand score was 9.1 points.

**Figure 2.**
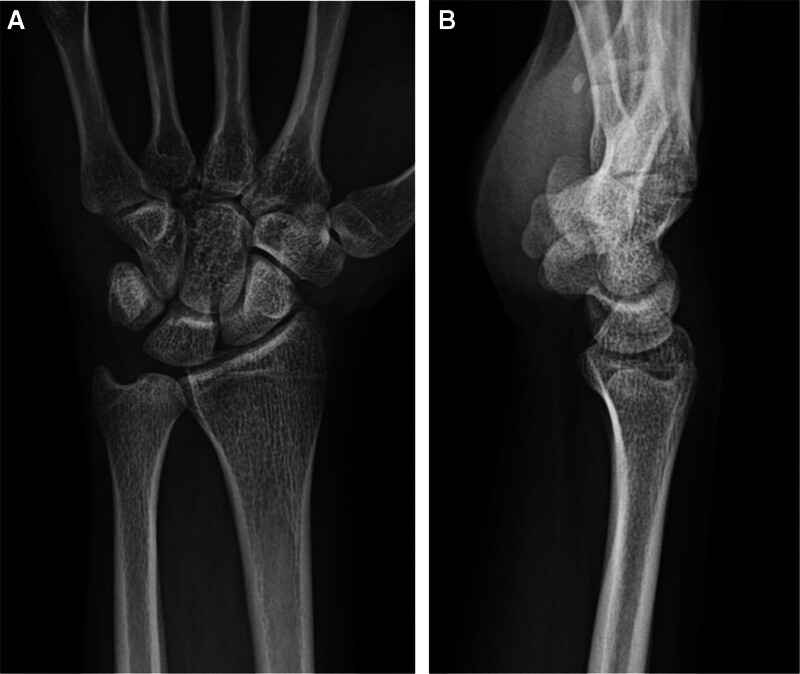
(A, B). Initial plain radiographs of the patient. Posterior-anterior (A) and lateral (B) View showed no abnormalities.

**Figure 3.**
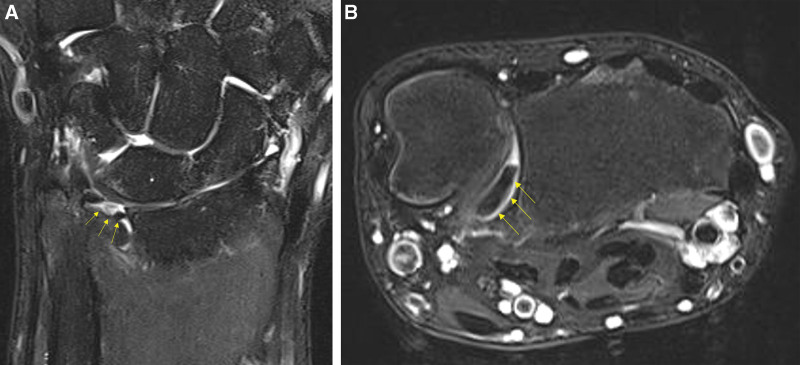
(A, B). T2-weighted magnetic resonance imaging (MRI) of the patient. In coronal view (A) it shows high signal intensity change and discontinuity in the triangular fibrocartilage complex (TFCC) central portion suggesting TFCC central and radial side tear (arrows). In axial view (B) it shows a torn TFCC flap is interposed in the distal radioulnar joint (DRUJ) at the volar side (arrows).

The patient underwent several diagnostic imaging tests. Plain radiographs showed no abnormalities, distal forearm bone fractures, ulnar subluxation, or DRUJ widening (Fig. 2A and B). However, MRI revealed some specific findings. We identified a tear in the central portion of TFCC on T2-weighted MRI. The torn flap of the TFCC was interposed at the volar side of the DRUJ (Fig. 3A and B).

**Figure 4.**
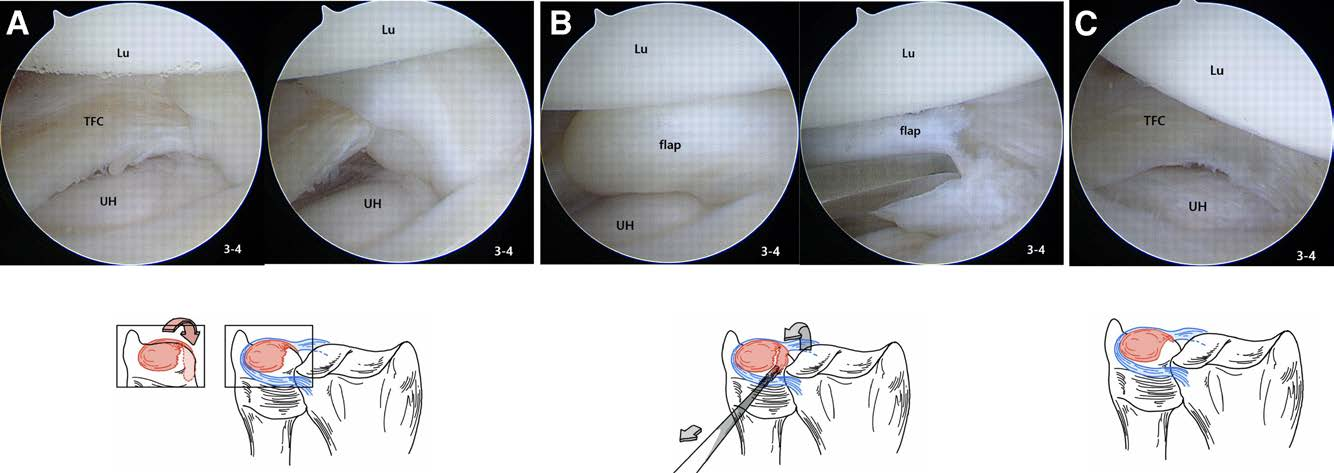
Arthroscopic findings and illustrations of Lt. wrist joint. (A) The left upper arthroscopic view shows the TFCC central portion tear and the right upper arthroscopic view shows the interposition of its flap in the volar side of the ulnar head in DRUJ. The lower illustration represents the schematic diagram of the arthroscopic findings mentioned above. (B) The left upper arthroscopic view shows a reduction of the flap. The right upper arthroscopic view shows partial resection of the flap. The lower illustration represents the schematic diagram of the arthroscopic procedures mentioned above. (C) Upper arthroscopic view and lower illustration represent the postresection and debridement state of the TFCC. DRUJ = distal radioulnar joint, TFCC = triangular fibrocartilage complex.

The patient underwent arthroscopic surgery on the left wrist joint. Arthroscopic examination revealed a tear in the TFCC on the radial side. The torn flap was approximately 1.5 cm in size and was interposed at the volar side of the DRUJ. This resulted in DRUJ locking with supination limitation. We removed the flap from the DRUJ using an arthroscopic grasper and partially resected it (Fig. 4A–C). After removing the arthroscopic instrument, we tested the forearm ROM. There was no locking and the forearm was well supinated.

**Figure 5.**
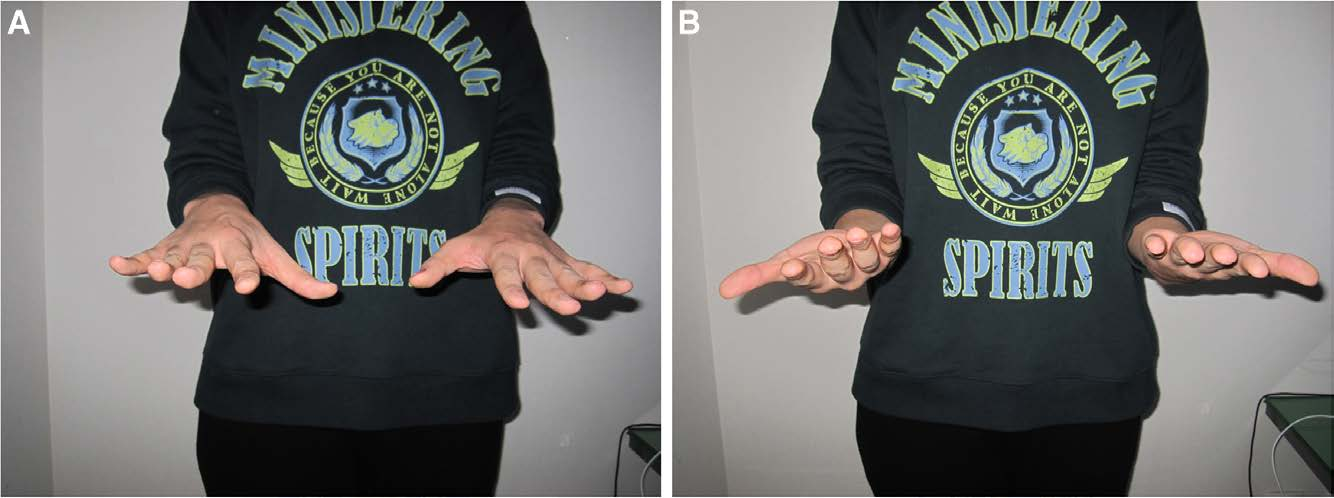
(A, B). Postoperative gross photo for ROM of pronation (A) and supination (B) in both forearms. It shows equal full ROM in both forearms. ROM = range of motion.

Two months after the surgery, the patient had no pain and showed full forearm supination (Fig. 5A and B). He showed equal grip strength in both hands; 30 kg strength in both hands. Visual analog scale score was 0 point. The Quick Disabilities of Arm, Shoulder and Hand score was 0 points. Twelve months after surgery, we interviewed the patient over the phone and stated that there was no more pain or limitation of ROM in the left wrist joint.

## 3. Discussion

Acute blockage of forearm supination can occur after minor trauma. This could be due to DRUJ injuries in relatively young patients engaging in leisure activities.^[1,2]^ Several studies have suggested blockage of volar translation of the ulnar head during supination due to TFCC rupture as the mechanism of forearm supination blockage.^[1–6]^ Suzuki et al^[1]^ compared 24 cases of acute blockage of forearm supination due to TFCC rupture and presented the location and blocking mechanism of TFCC injuries in 3 categories. First, in the case of dorsal DRUJ injury, the torn portion is deviated and inserted into the volar side of the ulnar head, blocking its translation of the ulnar head. Second, in the case of DRUJ volar portion injury, the torn portion becomes stuck under the volar aspect of the ulnar head and blocks the volar motion of the ulnar head during supination, causing a limitation. Third, when a TFCC tear occurs in the form of a flap, kinking occurs between the volar side capsule of the DRUJ and the ulnar head, causing DRUJ locking. Of the 24 reported cases, 15 were dorsal injuries, 8 were volar injuries, and 1 was a flap tear. Additionally, the authors recommended arthroscopic reduction, because manual reduction may cause additional tears. In our case, the patient showed supination limitation but no pronation limitation, or DRUJ instability. Arthroscopically, the torn flap was kinked between the ulnar head and volar DRUJ capsule. Based on the above classification, it was classified as a flap tear. In addition, Suzuki et al^[1]^ confirmed the diagnosis using an arthrogram and reported that MRI may not be helpful. However, in our case, the specific finding of a TFCC injury with the torn flap was confirmed on MRI.

After removing the torn flap through arthroscopic surgery, the patient symptoms improved, as evidenced by physical examination and several scores. Therefore, we suggest that when patients complain of acute blockage of forearm supination after minor trauma, physicians should suspect DRUJ blocking due to a TFCC injury. In addition, when patients are diagnosed with supination limitation due to TFCC injury, we recommend treatment through arthroscopic surgery rather than manual reduction because arthroscopic surgery can confirm the diagnosis with evaluation and classification, and it presents a more precise reduction without additional injury than manual reduction.

Our case report had several limitations. First, the patient minor trauma was confirmed, but considering his symptoms and duration between onset and first hospital visit, additional investigation is needed to determine whether the TFCC tear was due to repeated minor injuries. In addition, a long-term follow-up was not achieved although there were no problems until the last follow-up. However, we conducted a phone interview with him 12 months after surgery, and he still had no problems.

## 4. Conclusion

Acute blockage of the forearm supination is rare. When it occurs after minor trauma, DRUJ blocking due to a TFCC injury should be suspected. In this case, MRI can help in diagnosis by identifying TFCC tears. Diagnosis should be confirmed by directly checking for DRUJ locking caused by a tear on arthroscopy. Reduction and debridement of the torn flap through arthroscopic surgery are recommended for treatment.

## Author contributions

**Conceptualization:** Young-Keun Lee.

**Data curation:** Jee Yune Kim, Young-Keun Lee.

**Formal analysis:** Young-Keun Lee.

**Investigation:** Ji Woong Ho, Jee Yune Kim, Young-Keun Lee.

**Methodology:** Young-Keun Lee.

**Software:** Jee Yune Kim.

**Supervision:** Young-Keun Lee.

**Writing – original draft:** Ji Woong Ho, Jee Yune Kim, Young-Keun Lee.

**Writing – review & editing:** Ji Woong Ho, Young-Keun Lee.
